# Predictive value of elastography (Shear-Wave and Elasticity Index) in differentiating benign from malignant breast lesions: a retrospective study in Peru

**DOI:** 10.2478/raon-2026-0018

**Published:** 2026-04-16

**Authors:** Gaston (Wilmer) Mendoza, Nancy Muñoz, Jorge Aguilar, Jorge Ayón, Diana Díaz-Llontop, Samantha Mendoza, Claudia Quiñones, Richard Castillo, Luis Taxa

**Affiliations:** 1 Detecta Clinica, Lima, Peru. Av. Angamos Este 2688 - Surquillo, Lima, Peru; 2 Universidad San Martín de Porres, Lima, Peru. Av. Alameda del Corregidor 1531, La Molina, Lima

**Keywords:** breast elastography, shear wave elastography, elasticity index, BI-RADS, breast neoplasms, diagnostic ultrasonography, Peru

## Abstract

**Background:**

Evidence on the usefulness of quantitative elastography in the characterization of breast lesions remains limited in Latin America and scarce in Peru. This study aims to fill a relevant scientific gap by establishing specific cut-off points to optimize diagnostic interpretation and improve accuracy in differentiating benign from malignant lesions.

**Patients and methods:**

An observational, retrospective, and analytical study was conducted in 189 patients who underwent breast elastography between October 2024 and September 2025. Clinical and radiological variables were analysed. Bivariate tests and multivariate binary logistic regression were applied to identify independent predictors of malignancy. The cut-off points were determined *via* receiver operating characteristic (ROC) curve analysis.

**Results:**

Of the 85 lesions with histopathological confirmation, 54 (63.5%) were malignant, and 31 (36.5%) were benign. Malignant lesions presented significantly greater values of the elastography index (2.5 *vs*. 1.9; p = 0.001) and shear-wave elastography (SWE) (53 *vs*. 43 kPa; p < 0.001). According to the adjusted model, the Breast Imaging Reporting and Data System (BI-RADS) category remained the most relevant radiological predictor, suggesting that elastography values can be considered complementary for more accurate classification. The optimal SWE cut-off value of ≥ 47.5 kPa demonstrated acceptable diagnostic performance, with a sensitivity of 79.6% and specificity of 67.7%, indicating an adequate ability to distinguish between benign and malignant breast lesions.

**Conclusions:**

This study represents one of the first examples of Peruvian evidence contributing scientific information on the application of quantitative elastography in the evaluation of breast lesions.

## Introduction

Breast cancer is the most common malignancy and one of the leading causes of cancer-related death among women worldwide and in Peru.^1–4^ Despite advances in early diagnosis, its incidence and mortality rates continue to rise, underscoring the need to optimize complementary diagnostic strategies for accurate clinical decision-making.

Although digital mammography remains the standard screening method, its diagnostic performance may be affected by breast density, with sensitivity potentially decreasing to 70%.^5–7^ In such cases, breast ultrasound serves as a useful complementary tool that enhances the detection of lesions not visible via mammography.^8^

Ultrasound elastography has emerged as a noninvasive technique that quantifies tissue stiffness and improves the characterization of breast lesions.^9^ Among its modalities, stands out for providing quantitative parameters such as the mean elasticity value (Emean) and the elasticity index (E-ratio), which reflect biomechanical differences between benign and malignant tissues.^9–11^ Integrating these parameters into the Breast Imaging Reporting and Data System (BI-RADS) system has been shown to increase diagnostic specificity, particularly in intermediate categories (BI-RADS 4A and 4B).^10–12^

However, the proposed cut-off values for SWE and the elasticity index vary widely across studies, with thresholds ranging from 45 to 90 kPa or E-ratio values between 2.3 and 4.8, largely due to differences in equipment, protocols, and population characteristics.^7^,^11–18^ This lack of standardization represents a significant scientific gap that limits the clinical application of this technique in Peru. International guidelines (EFSUMB and WFUMB) recommend that each centre or population establish its own reference values on the basis of local evidence, adjusted to demographic and technical characteristics.^13,18^

In Latin America, evidence remains limited, and in Peru, specific cut-off points for quantitative elastography have not yet been established. This scientific gap restricts the full incorporation of this technology into national clinical practice.^19–21^ In this context, the present study seeks to address this gap by evaluating the predictive value of the elasticity index and shear wave elastography (SWE) in differentiating benign and malignant breast lesions classified as BI-RADS 4A, 4B, 4C, and 5 and by proposing cut-off points adapted to the Peruvian population to support more precise clinical decision-making.

## Patients and methods

A retrospective review was conducted via an anonymized database that included the clinical and pathological data of patients who underwent elastography at Detecta Clínica. Data collected between October 2024 and September 2025 were analysed, including variables such as age, family history of cancer, ultrasound tumor size, ultrasound lymph node size, ultrasound BI-RADS category, elasticity index, SWE (kPa), and confirmed diagnosis. All patients were evaluated according to the institution’s standard clinical practice.

Ultrasound findings from two different elastography techniques (Elastography Index and Shear Wave Elastography [SWE]) will be evaluated in all patients *via* a *Resona 7 MINDRAY* system equipped with a linear transducer L14-5 and software for tumor shell (capsule) measurement and data storage.

All information was obtained from the institutional medical records of Detecta Clinica as part of a retrospective review of routinely recorded clinical data.

### Inclusion and exclusion criteria

All consecutive patients who underwent breast elastography during the study period were eligible for inclusion. However, due to the retrospective nature of the study, only patients with histopathological results available in the institutional electronic health record were included in the diagnostic accuracy analysis. Histopathological confirmation was available only for lesions biopsied or surgically excised at our institution. Lesions without available biopsy results in our database were excluded from analyses requiring histological verification, as biopsy may have been performed at external institutions or data were not accessible in the institutional records.

### Detailed description of the shear wave elastography (SWE) protocol and elasticity index calculation

Quantitative SWE was performed *via* a *Mindray* ultrasound system equipped with a sound-touch elastography (STE) module and a linear transducer dedicated to breast examinations. The examination was carried out with the patient in the supine position and minimal probe compression. The breast examination mode was selected, followed by activation of the “Elasto” function and selection of “STE” on the touchscreen interface. Patients were instructed to hold their breath during image acquisition to minimize motion artefacts.

A region of interest (ROI) was positioned over the lesion via the trackball, adjusting its size as necessary via the touchscreen and control knob. The *HQElasto* mode was activated to generate a high-quality, two-dimensional colour map representing the propagation velocity of shear waves across the tissue. Image stability was confirmed when the M-STB indicator displayed five green stars and when the M-RLB index exceeded 80%, ensuring optimal acquisition quality.

After stabilization, the image was captured by pressing UPDATE, and the “Measure” function was selected. The operator chose Elasticity/Mass *Elas*. mode and defined the shell size for measurement. The system automatically calculates the elasticity index (elinary index) in kilopascals (kPa), which is derived from the mean shear wave velocity within the ROI according to the following formula:

$$E=3\rho v^{2}$$

where E is the elasticity modulus (kPa), ϱ is the tissue density (assumed to be 1,000 kg/m^3^), and v is the shear wave velocity (m/s).

Each measurement was saved individually via the SAVE function, and a summary report was generated via the Info/Review menu.

Shear wave elastography (SWE) values were automatically generated by the ultrasound system. The transducer produced acoustic radiation force impulses to induce shear waves within the lesion, and the system measured the resulting shear wave propagation velocity (m/s) inside the ROI. On the basis of the physical model of nearly incompressible soft tissue, the device converted these velocities into elasticity values expressed in kilopascals (kPa) via the manufacturer’s algorithm, derived from the standard relationship. For each injury, two measurements were taken and averaged to obtain the representative SWE value.

For each lesion, the elastography software automatically provided quantitative stiffness parameters within the region of interest, including mean, maximum, minimum, and standard deviation values expressed in kilopascals (kPa). For statistical analysis, the mean SWE value (Emean) was selected as the representative measurement, as it reflects the overall stiffness of the lesion and reduces the influence of focal extreme values.

### Statistical analysis

The study design was observational, retrospective, and analytical. Data analysis was performed via SPSS software, version 22.0.

A descriptive analysis was conducted to determine the distribution of each variable, followed by a bivariate analysis to explore associations between clinicopathological variables and the SWE elasticity index. Finally, a multivariate analysis was performed to evaluate the relationships between clinicopathological factors and elastography results, adjusting for potential confounding variables.

Variables included in the multivariate logistic regression models were selected based on clinical relevance and prior evidence. An initial comprehensive model incorporated demographic factors (age), lesion characteristics (tumor size), elastography parameters (SWE and elastography index), and ultrasound BI-RADS category. Given the wellestablished predictive dominance of BI-RADS, additional clinically driven models were constructed by sequentially excluding BI-RADS and, subsequently, age, in order to assess the independent contribution of stiffness-based parameters. This stepwise approach was used to address potential collinearity between BI-RADS and elastography variables and to clarify their complementary diagnostic roles.

Optimal cut-off values for Shear-Wave Elastography (SWE) and the Elastography Index were determined using the Youden index, calculated as *(sensitivity + specificity - 1)*, which identifies the threshold that maximizes the combined diagnostic performance of both metrics. All possible cut-off values within the study cohort were evaluated, and the thresholds yielding the maximum Youden index were selected as optimal. Diagnostic performance was subsequently assessed at these cut-offs. Ninety-five percent confidence intervals (95% CI) for sensitivity and specificity were calculated using the exact Clopper-Pearson method.

### Ethical approval

This study was approved by the *“Comite Institucional de Bioética de Vía Libre* ”, which is registered with the Peruvian *Instituto Nacional de Salud*. Approval was granted under protocol number DETECTA-00004-2025, with a validity period from Oct 28, 2025 to Oct 27, 2026.

All study procedures complied with institutional policies on data confidentiality and secure handling of clinical information. The investigators were certified in good clinical practice and research ethics prior to conducting the study.

Given the retrospective and observational nature of the study - based exclusively on previously recorded and anonymized clinical data - the ethics committee formally waived the requirement for individual informed consent in accordance with the principles of the Declaration of Helsinki (2013 revision) and applicable Peruvian national research regulations.

## Results

### Descriptive analysis

A total of 189 patients who underwent breast elastography between October 2024 and September 2025 were included in the study. Among them, 55 (29.1%) had no prior mammography performed at the institution. On ultrasound evaluation, 43.4% of the lesions were classified as BI-RADS 5.

The median ultrasound tumor size was 20 mm (IQR 17–28), the median elasticity index was 2.57 (IQR 2–3), and the median SWE value was 54 kPa (IQR 49–59).

Among the 85 lesions with available histologic diagnoses, 54 (63.5%) were malignant, and 31 (36.5%) were benign. The most frequent molecular subtype was luminal B (62.9%), followed by luminal A (20.0%), HER2-positive (11.4%), and triplenegative (5.7%). The median histologic grade was 2 [IQR 2–3]. The general characteristics are summarized in Table 1.

**TABLE 1.** Clinical and radiological characteristics of the cohort

Variable	n (189)	%/Median (IQR)
Age (years)	58 (48-68)	—
Family history of cancer		
Yes	42	22.22
No	140	74.07
Not declared	7	3.70
Lesion laterality		
Right	83	43.90
Center	106	56.10
Previous mammography	189	
Yes	134	70.90
No	55	29.10
Ultrasound BI-RADS category		
4A	21	11.10
4B	70	37.00
4C	16	8.50
5	82	43.40
Ultrasound tumor size (mm)	20 (17-28)	—
Lesion depth	16 (12-20)	
Elasticity index	2.57 (2-3)	—
Shear-Wave Elastography (SWE, kPa)	54 (49-59)	—
Histopathological diagnosis	85	
Malignant	54	63.50
Benign	31	36.50
Breast cancer subtype	n = 35	
Luminal A	7	20.00
Luminal B	22	62.90
HER2-positive	4	11.40
Triple negative	2	5.70
Histological grade	2 (2-3)	—

1 Data are expressed as frequencies (%) or medians (interquartile ranges, IQRs), as appropriate.

1 BI-RADS = Breast Imaging Reporting and Data System; SWE = shear wave elastography

### Bivariate analysis

Normality was assessed via the Kolmogorov-Smirnov test. Elastography variables showed nonnormal distributions (elastography index: p = 0.021; SWE: p < 0.001). Lesion size and depth also fail to meet normality assumptions. Therefore, nonparametric tests were used for the bivariate analyses.

#### Correlation between lesion characteristics and elastography measurements

Spearman’s rho correlation analysis revealed that lesion size was positively associated with both elastography parameters. A weak but statistically significant correlation was observed between size and the elastography index (ϱ = 0.226, p = 0.002), as well as between size and the SWE value (ϱ = 0.250, p = 0.001). These findings indicate that larger breast lesions tend to demonstrate greater tissue stiffness, as measured by both elastography techniques.

Conversely, lesion depth was not significantly correlated with elastography measurements. The correlation coefficients were low and non-significant for both the elastography index (ϱ = –0.037, p = 0.622) and the SWE (ϱ = 0.102, p = 0.176), suggesting that tissue stiffness values are independent of lesion depth within the ranges evaluated. Differences in sample size reflect missing depth data in some radiology reports (Table 2).

**TABLE 2.** Spearman’s Rho correlation between lesion characteristics and elastography measurements

Rho de Spearman			Elasticity index	SWE (kPa)
**Lesion size (mm)**	Spearman’s ρ	1.000	0.226	0.250
	p - value		**0.002**	**0.001**
	N	189	188	189
**Lesion depth**	Spearman’s ρ	1.000	-0.037	0.102
	p - value		0.622	0.176
	N	177	176	177

1 Spearman’s rho correlation coefficients are shown for the associations between lesion size or lesion depth and elastography parameters. Values of p < 0.05 were considered statistically significant. The autocorrelation values (ρ = 1.000) correspond to the variable with itself.

1 **SWE =** shear wave elastography

### Comparison according to histopathological diagnosis

Malignant lesions presented significantly greater values for both the elasticity index and the SWE(Mann–Whitney U test, p < 0.05).

The median elasticity index was 2.5 [2.0–2.8] for malignant lesions and 1.9 [1.4–2.5] for benign lesions (p = 0.001).

The median SWE value was 53 [48–59] kPa for malignant lesions and 43 [36–49] kPa for benign lesions (p < 0.001) (Table 3).

**TABLE 3.** Comparison of elastography values according to histopathological diagnosis

Histopathological Diagnosis			
	**Benign (n = 31)** Median; [Q1-Q3]	**Malignant (n = 54)** Median; [Q1-Q3]	p value
Elasticity index	1.9; [1.4–2.5]	2.5; [2–2.8]	**0.001**
Shear-Wave Elastography (SWE, kPa)	43; [36–49]	53; [48–59]	**0.000**

1 Comparisons were performed via the Mann–Whitney U test (p < 0.05 was considered significant).

1 Me = median; Q1 = first quartile; Q3 = third quartile.

### Relationship between Breast Imaging Reporting and Data System (BI-RADS) and elastography values

A progressive and significant association was observed between the BI-RADS category and tissue stiffness quantified by elastography. The median SWE values increased gradually from 38 kPa in BI-RADS 4A to 59 kPa in BI-RADS 5, whereas the elasticity index rose from 1.6 to 2.7 across the same spectrum. Both associations were statistically significant (p = 0.001, Kruskal-Wallis test) (Table 4).

**TABLE 4.** Relationship between BI-RADS category and elastography measurements

Ultrasound BI-RADS	SWE (kPa) Median (IQR)	p value*	Elasticity index Median (IQR)	p value*
4A	38; [29.5–39.5]	**0.001**	1.6; [1.2–2.05]	**0.001**
4B	45.5; [39–50]		1.9; [1.5–2.20]	
4C	48; [38–55]		2.55; [2–2.80]	
5	59; [49–60]		2.7; [2.15–2.80]	

1 BI-RADS = Breast Imaging Reporting and Data System; IQR = interquartile range; Me = median; SWE = shear wave elastography.

* p value obtained via the Kruskal-Wallis test (p < 0.05 considered significant)

**FIGURE 1.**
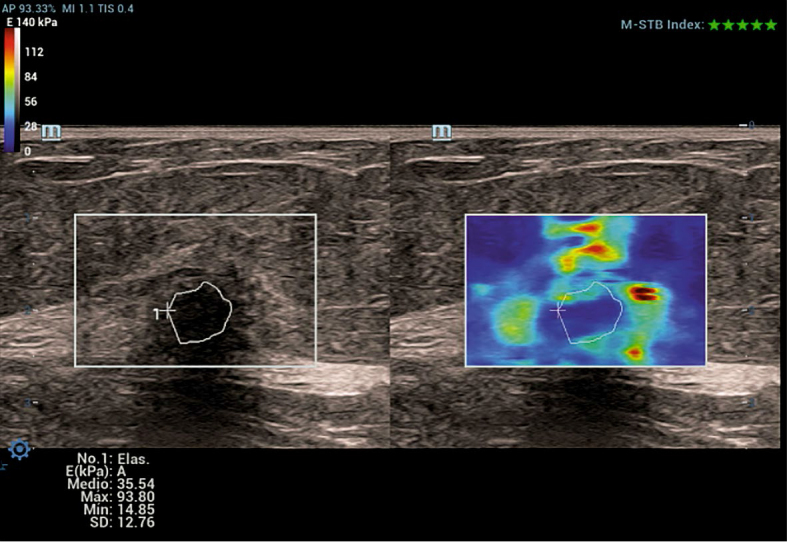
Representative shear-wave elastography image of a BI-RADS 4B breast lesion. Grayscale ultrasound image (left) and corresponding shear-wave elastography map (right) of a BI-RADS 4B breast lesion. The region of interest (ROI) is manually traced along the lesion margins on B-mode imaging. The elastography map shows intermediate stiffness with focal areas of increased rigidity surrounding the lesion, depicted by green to yellow colour coding, without the marked high-stiffness dominance seen in higher-suspicion categories. Quantitative measurements demonstrate a moderate mean SWE value (Emean ≈ 35.5 kPa), illustrating the role of SWE in refining risk stratification within intermediate-suspicion BI-RADS subcategories. Individual SWE values may vary within BI-RADS subcategories; this representative BI-RADS 4B case illustrates moderate mean stiffness with focal areas of increased rigidity, consistent with the heterogeneous nature of this category.

**FIGURE 2.**
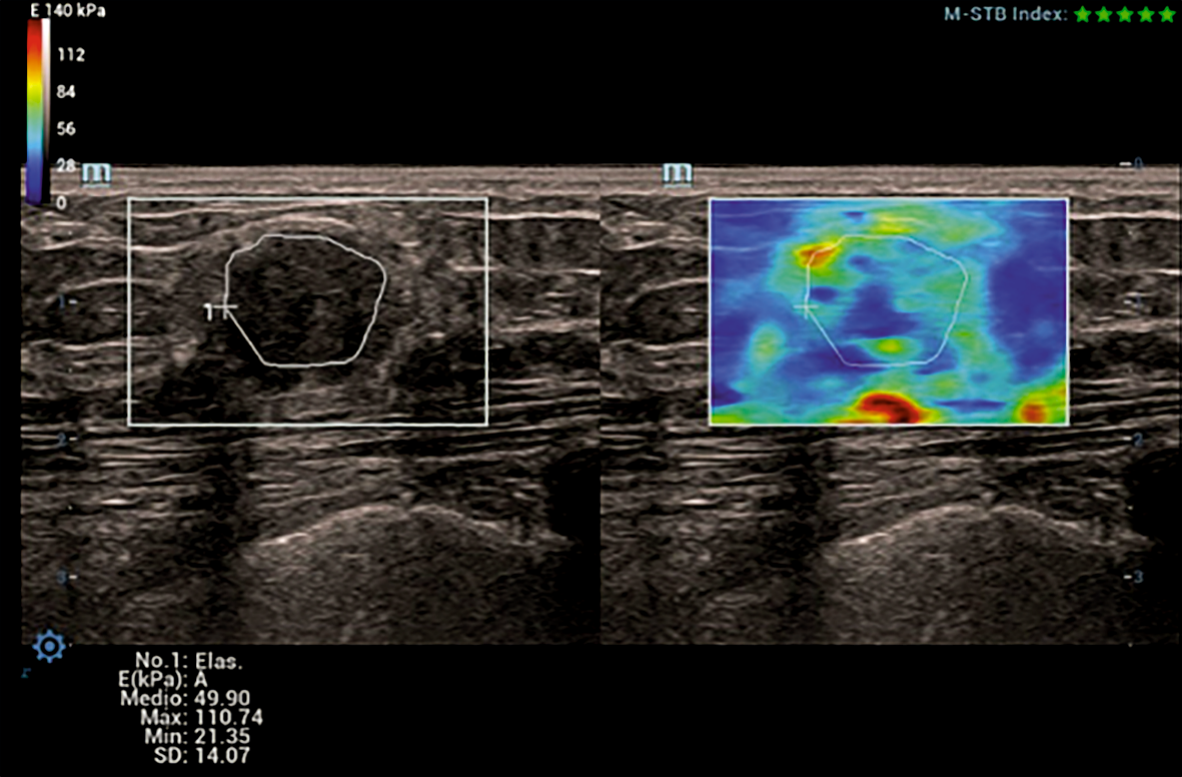
Representative shear-wave elastography image of a BI-RADS 4C breast lesion. Grayscale ultrasound (left) and corresponding shear-wave elastography (right) of a BI-RADS 4C breast lesion. The region of interest (ROI) is manually delineated along the lesion margins. The elastography map demonstrates marked stiffness heterogeneity with focal areas of increased rigidity (red–orange colour scale). Quantitative measurements show an elevated mean SWE value (Emean ≈ 49.9 kPa) and high maximum stiffness, findings consistent with a high suspicion for malignancy and illustrating the added diagnostic value of SWE in advanced BI-RADS subcategories.

Representative ultrasound and shear-wave elastography images are provided to visually illustrate the relationship between BI-RADS subcategories and quantitative stiffness values, highlighting the progressive increase in tissue rigidity from intermediate-to high-suspicion lesions (Figure 1, 2).

### Relationships between the elastography values and BI-RADS subcategories

A comparative analysis was performed to evaluate the elastography measurements across BI-RADS 4A and 4B lesions. The median SWE values were significantly greater in BI-RADS 4B lesions (45 kPa; IQR: 39–50) than in BI-RADS 4A lesions (38 kPa; IQR: 29.5–39.5), with a highly significant difference (p < 0.001, Mann-Whitney U test). This finding highlights the strong discriminatory ability of SWE to differentiate between these adjacent subcategories, reflecting the greater stiffness typically associated with lesions of greater suspicion.

Similarly, the elastography index was significantly different between the groups, although with a smaller effect size. BI-RADS 4B lesions presented higher median values (1.90; IQR: 1.50–2.20) than BI-RADS 4A lesions did (1.60; IQR: 1.20–2.05) (p = 0.030). While both elastography parameters contribute to distinguishing between BI-RADS categories 4A and 4B, SWE exhibited superior discriminative performance, supporting its use as a more robust stiffness-based indicator in the stratification of intermediate-suspicion breast lesions (Table 5).

**TABLE 5.** Elastography measurements according to BI-RADS 4A and 4B subcategories

Parameter	BI-RADS 4a Median [Q1-Q3]	BI-RADS 4b Median [Q1-Q3]	p value*
Shear-Wave Elastography (SWE), kPa	38.0 [29.5–39.5]	45.0 [39.0–50.0]	< 0.001
Elastography Index	1.60 [1.20–2.05]	1.90 [1.50–2.20]	0.030

1 Q1 = first quartile; Q3 = third quartile;

* = Statistical test: Mann–Whitney U test

### Correlation between tumor size and tissue stiffness

A weak but significant positive correlation was identified between ultrasound tumor size and elastography values:

- Elasticity index: ϱ = 0.226; p = 0.002
- SWE: ϱ = 0.250; p = 0.001

Tumor size explained 5.1% of the variability in the elasticity index and 6.3% of the variability in SWE, indicating that while size influences tissue stiffness, it does not primarily determine malignancy (Table 6).

**TABLE 6.** Spearman correlation between tumor size and elastography parameters

Parameter	Spearman’s ρ	p value	R^2^ (%)
Elastography Index	0.226	0.002	5.1
Shear-Wave Elastography (SWE, kPa)	0.250	0.001	6.3

1 Spearman’s rank correlation test. R^2^ represents the percentage of variability explained by tumor size. p < 0.05 was considered statistically significant.

### Multivariate analysis

A multivariate binary logistic regression model was performed to identify independent predictors of malignancy among the clinical and imaging variables (Table 5). After adjustment, age and BIRADS category remained statistically significant predictors, whereas SWE and the elastography index demonstrated attenuated associations, suggesting a complementary—rather than primary— diagnostic contribution.

Age had a modest but significant effect: each one-year increase was associated with a 10.8% increase in the odds of malignancy (adjusted odds ratio obtained from the multivariate logistic regression model [ORa]= 1.108, 95% CI: 1.033-1.187; p = 0.004). Tumor size did not retain statistical significance in the adjusted model (ORa = 0.967, 95% CI: 0.928-1.009), indicating that lesion dimensions alone were not strong independent predictors once the model accounted for elastography and BIRADS descriptors.

For the elastography parameters, the crude (unadjusted) model revealed that both the SWE and elastography indices were strongly associated with malignancy. Each 1 kPa increment in SWE increased the odds by 11% (unadjusted crude odds ratio [ORc] = 1.107, 95% CI: 1.047–1.171), and a one-unit increase in the elastography index increased the odds more than threefold (ORc = 3.59, 95% CI: 1.65–7.81). However, in the adjusted model, the strength of these associations decreased (SWE ORa = 0.846, 95% CI: 0.242–2.958; elastography index ORa = 1.085, 95% CI: 0.983–1.198), and neither remained statistically significant.

This attenuation indicates that while elastography parameters correlate with malignancy, their predictive value overlaps substantially with that of BI-RADS, which integrates multiple morphological features and therefore carries superior diagnostic weight.

Because BI-RADS integrates multiple morphological ultrasound features, including shape, margins, orientation, and posterior acoustic behaviour, partial overlap with stiffness-related information derived from elastography is expected. This overlap may result in collinearity and attenuation of elastography effects in fully adjusted models. Theuse of alternative regression models excluding BIRADS allowed clearer identification of the independent predictive value of SWE.

Among all the variables included, the ultrasound BI-RADS category remained the strongest independent predictor of malignancy, even after adjustment (ORa = 2.93, 95% CI: 1.34–6.40; p = 0.007). This finding reinforces the importance of the BI-RADS score as the dominant radiological variable in risk stratification. Importantly, the persistence of a positive trend in the elastography parameters suggests that SWE and the elasticity index provide complementary diagnostic information, supporting their value as adjunct tools that increase confidence, refine risk assessment, and potentially improve discrimination in borderline cases such as BI-RADS 4 subcategories (Table 7).

**TABLE 7.** Binary logistic regression for predictors of malignancy

Parameter	B	*p* value	ORc (95% CI)	*p* value	ORa (95% CI)
Age (years)	0.110	< 0.001	1.117 (1.060–1.176)	0.004	1.108 (1.033–1.187)
Tumor size (mm)	–0.025	0.074	0.976 (0.949–1.002)	0.122	0.967 (0.928–1.009)
Shear-Wave left	0.102	< 0.001	1.107 (1.047–1.171)	0.793	0.846 (0.242–2.958)
Elastography Index	1.278	0.001	3.588 (1.648–7.813)	0.106	1.085 (0.983–1.198)
Ultrasound BI-RADS	1.459	< 0.001	4.304 (2.411–7.683)	0.007	2.932 (1.343–6.400)

1 Binary logistic regression model. p < 0.05 was considered statistically significant

1 CI = confidence interval; ORa = adjusted odds ratio obtained from the multivariate logistic regression model; ORc = crude odds ratio (unadjusted).

### Logistic regression analysis based on clinically selected variables

A secondary logistic regression analysis was conducted to explore the independent predictive value of the elastography parameters after BI-RADS was removed from the model. Given that BI-RADS is a well-established and highly dominant predictor of malignancy, its inclusion can overshadow or suppress the effects of other covariates. Therefore, BI-RADS was intentionally excluded to evaluate whether quantitative elastography provides additional diagnostic information beyond standard morphological classification.

### Model including age, elastography index, and shear wave elastography (SWE)

In the first model, the variables retained were age, the elastography index, and SWE (kPa) (Table 8).

**TABLE 8.** Binary logistic regression model using clinically selected variables

Parameter	B	p value	Adjusted OR (ORa)	95% CI (Lower)	95% CI (Upper)
Age (years)	0.110	< 0.001	1.117	1.054	1.783
Elastography Index	0.422	0.435	1.524	0.530	4.389
SWE (kPa)	0.094	0.016	1.099	1.017	1.186

1 CI = confidence interval; ORa = adjusted odds ratio; p < 0.05 was considered statistically significant

Age remained a significant predictor: each additional year increased the probability of malignancy by 11.7% (ORa = 1.117, 95% CI: 1.054–1.783; p < 0.001), confirming its consistent contribution across models. SWE demonstrated a statistically significant association with malignancy (ORa = 1.099, 95% CI: 1.017–1.186; p = 0.016), indicating that for every 1 kPa increase in stiffness, the odds of malignancy increased by approximately 10%. This finding reinforces the strong discriminatory capacity of SWE even when BI-RADS is excluded from the model. In contrast, the elastography index did not reach statistical significance (ORa = 1.524, 95% CI: 0.530–4.389; p = 0.435). Although its direction of association was positive, the wide confidence interval and non-significant p value suggest that this parameter has lower independent predictive strength than does SWE.

Overall, SWE emerged as the predominant elastography predictor, retaining statistical significance, whereas the elastography index did not.

### Model excluding age

To assess whether age influences the relative performance of the elastography parameters, a reduced model was constructed that included only SWE and the elastography index (Table 9).

**TABLE 9.** Binary logistic regression model excluding age

Parameter	B	p value	Adjusted OR (ORa)	95% CI (Lower)	95% CI (Upper)
Elastography Index	0.671	0.117	1.955	0.846	4.521
SWE (kPa)	0.079	0.011	1.082	1.018	1.150

1 CI = confidence interval; ORa = adjusted odds ratio; SWE = shear wave elastography

1 p < 0.05 was considered statistically significant

**TABLE 10.** Area under the ROC curve (AUC) for elastography parameters

Parameter	AUC	SE	p value*	95% CI (Lower-Upper)
Elastog raphy Index	0.725	0.059	0.001	0.610-0.840
SWE (kPa)	0.768	0.056	< 0.001	0.659-0.877

1 p values were derived from the DeLong test. Statistical significance was defined as p < 0.05.

1 SWE = shear wave elastography

**TABLE 11.** Bootstrap validation of optimal cut-off points for elastography parameters

Method	Original cut-off	Bootstrap mean	Bootstrap 95% CI	Coefficient of Variation
SWE (kPa)	47.5	47.49	40.5-51.5	4.7%
Elastography Index	2.25	2.054	1.6-2.367	13.0%

1 Cut-off points were derived from the Youden index and validated via 1,000 bootstrap iterations. Lower coefficients of variation indicate more stable and reproducible thresholds.

1 SWE = shear wave elastography

**TABLE 12.** Diagnostic performance of elastography parameters

SWE	Diagnostic		Sensitivity (%)	Specificity (%)	PPV(%)	N PV(%)
	Malignant (n = 54)	Benign (n = 31)				
≥ 47.50 kPa	43	10	79.6	67.7	81.1	65.6
< 47.50 kPa	11	21				
**Elasticity index**						
≥ 2.25	36	9	66.7	71.0	80.0	55.0
< 2.25	18	22				
Total	54	31				

1 PPV = positive predictive value; NPV = negative predictive value; SWE = shear wave elastography

1 The cut-off values were determined via the Youden index.

In this simplified model, the elastography index remained non-significant (ORa = 1.955, 95% CI: 0.846–4.521; p = 0.117), and its effect size remained modest. In contrast, SWE was significantly associated with malignancy (ORa = 1.082, 95% CI: 1.0181–150; p = 0.011). Importantly, the magnitude and precision of the SWE effect were preserved even after age was removed, indicating the robustness of this predictor across model structures.

Across all the models tested, SWE consistently exhibited independent and statistically significant predictive value, whereas the elastography index did not achieve significance in any adjusted analysis. These results indicate that SWE is the most reliable quantitative elastography parameter for predicting malignancy when BI-RADS is excluded from the model. This finding supports its use as the preferred stiffness-based adjunct to conventional ultrasound evaluation, offering incremental diagnostic value, particularly in intermediate-suspicion lesions.

### Diagnostic performance and cut-off points

The diagnostic performance of both elastography parameters was evaluated via receiver operating characteristic (ROC) curve analysis (Figure 1). The ROC curves for SWE (blue line) and the elastography index (red line) demonstrated that both techniques discriminate between benign and malignant breast lesions, as both curves lie well above the diagonal reference line. This finding indicates that performance is superior to chance.

Compared with the elastography index, SWE exhibited a consistently greater trajectory across most of the ROC space, suggesting a more favourable balance between sensitivity and specificity throughout the range of possible thresholds. The curve for SWE also showed a smoother and more stable ascent, reflecting more consistent diagnostic behaviour across cut-off values, whereas the elastography index curve displayed minor irregu-larities, indicating greater variability at different threshold levels.

These visual differences were quantitatively corroborated. The area under the curve (AUC) for SWE was 0.768, indicating good discriminative ability (AUC 0.7–0.8). The elastography index achieved an AUC of 0.725, which also falls within the moderate-to-good range but is clearly lower than that of SWE. This separation was most pronounced in the middle of the range of false-positive rates, where SWE maintained higher sensitivity values for comparable specificity levels.

**FIGURE 3.**
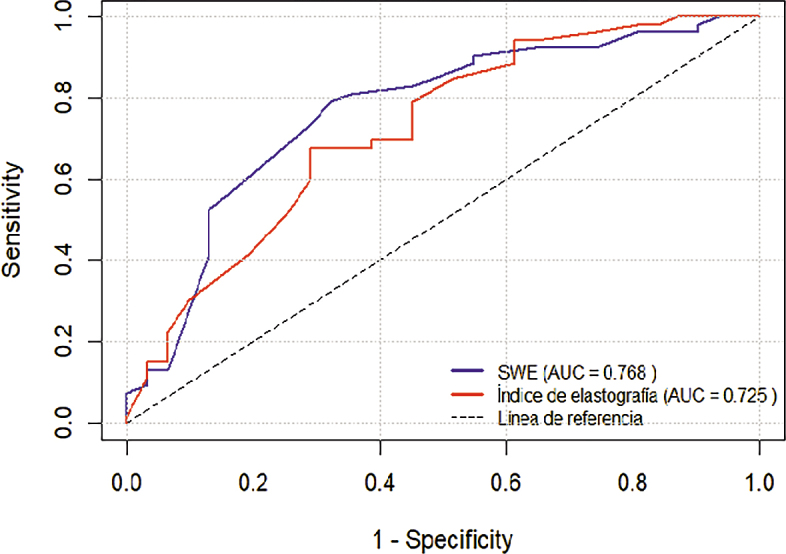
Receiver operating characteristic (ROC) curves for shear wave elastography (SWE) and the elastography index. ROC curves comparing the diagnostic performance of SWE (blue line) and the elastography index (red line) for distinguishing malignant from benign breast lesions. The diagonal dashed line represents the reference line of no discrimination. Both parameters demonstrated discriminative ability above chance, with SWE showing a greater area under the curve (AUC = 0.768) than the elastography index (AUC = 0.725), indicating superior overall diagnostic performance.

Although both techniques demonstrated clinical usefulness, SWE provided superior global discriminative performance, which was consistent with its higher AUC (Figure 3).

### Area under the curve (AUC)

Although the AUC for SWE was greater than that for the elastography index, the DeLong test did not reveal a statistically significant difference between the two curves, indicating comparable clinical utility. Both AUC values exceeded 0.70, confirming acceptable accuracy, with SWE trending toward better performance.

### Determination and validation of the optimal cut-off points

Using the Youden index, the optimal cut-off values were identified as ≥47.5 kPa for SWE and ≥2.25 for the Elastography Index. At the SWE cut-off of ≥47.5 kPa, sensitivity was 79.6% (95% CI: 66.289.1%; 43/54 malignant lesions correctly identified), and specificity was 67.7% (95% CI: 48.8–82.8%; 21/31 benign lesions correctly classified). For the Elastography Index cut-off of ≥2.25, sensitivity reached 66.7% (95% CI: 52.5–78.5%; 36/54), while specificity was 71.0% (95% CI: 52.0–85.3%; 22/31). These findings confirm that SWE achieved higher sensitivity, whereas the Elastography Index demonstrated slightly higher specificity, supporting their complementary diagnostic roles.

The optimal cut-off points were calculated via the Youden index, which identifies the threshold that maximizes the combined sensitivity and specificity.

- Elastography index optimal cut-off: 2.25
  Sensitivity: 66.7%
  Specificity: 71.0%
- SWE optimal cut-off: 47.5 kPa
  Sensitivity: 79.6%
  Specificity: 67.7%

Consistent with the ROC analysis, SWE achieved a better balance between true-positive and false-positive rates, particularly within the BIRADS 4A–4B spectrum, where diagnostic refinement is most valuable.

### Bootstrap validation

To assess the stability of the cut-off points, a bootstrap resampling procedure (1,000 iterations) was performed. This approach simulates multiple repetitions of the study by repeated sampling with replacement from the original dataset, generating distributions of cut-off values.

Bootstrap analysis demonstrated that the SWE cut-off was highly stable (CV 4.7%), whereas the elastography index showed moderate variability (CV 13%). These findings further support the use of SWE as a more robust and reproducible parameter for lesion characterization in this population.

### Diagnostic accuracy metrics

The diagnostic performance metrics associated with the optimal cut-off values are presented in Table 6.

- SWE ≥ 47.5 kPa: sensitivity, 79.6%; specificity, 67.7%; PPV, 81.1%; NPV, 65.6%.
- Elasticity index ≥ 2.25: Sensitivity, 66.7%; specificity, 71.0%; PPV, 80.0%; NPV, 55.0%.

SWE demonstrated superior sensitivity and PPV, indicating better capacity for identifying malignant lesions and reducing false negatives. Although the elastography index achieved slightly higher specificity, its lower sensitivity and NPV suggest more limited utility as a standalone discriminator.

Together, the ROC curve, AUC, cut-off analyses, and bootstrap validation consistently demonstrated that SWE offers a superior diagnostic profile compared with the elastography index, particularly in enhancing discrimination among BI-RADS 4A-4B lesions. Although both techniques have clinical value, SWE provides more stable, reproducible, and diagnostically powerful information, reinforcing its role as the preferred quantitative elastography parameter in breast lesion assessment.

## Discussion

In this study, which included a cohort of 189 patients, both SWE values—expressed in kilopascals (kPa)—and the elasticity index were shown to significantly differentiate between benign and malignant breast lesions classified as BI-RADS 4A–5. These findings confirm the usefulness of quantitative elastography as a complementary tool to conventional ultrasound in the characterization of suspicious breast lesions.

Our results are consistent with international evidence. Huang *et al*. evaluated 280 patients via SWE and reported that elastic heterogeneity analysis achieved an AUC of 0.963, which was greater than that obtained with the mean or maximum values alone, with a sensitivity of 93.8%.^22^ Lin *et al*. reported in a Chinese population that incorporating the maximum stiffness value (Emax) into the BI-RADS system increased specificity without compromising sensitivity, reinforcing the role of SWE as a combined diagnostic tool.^23^ Similarly, Chen *et al*. analysed anisotropy in elastography and demonstrated that quantitative stiffness parameters outperform orientation-dependent indices, although transducer positioning can influence measurements.^23^ In small lesions, Guzmán-Aroca *et al*. reported that SWE maintains excellent discriminative ability, with a sensitivity of 75% and specificity of 98.5% in solid nodules smaller than 1 cm, highlighting its applicability even in subcentimeter tumors.^24^ Taken together, these findings reinforce that the correlation between tissue stiffness and malignancy, as well as its progressive gradient with increasing BI-RADS category, is consistent with patterns described in the international literature.

In Peru, although no studies have yet been published in high-impact indexed journals that integrate SWE into the BI-RADS system with clearly defined performance parameters, promising exploratory research has been conducted. Arroyo *et al*. (Pontificia Universidad Católica del Perú) reported mean stiffness values of 39.4 ± 12.0 kPa in benign lesions, 55.4 ± 7.0 kPa in malignant lesions, and 23.9 ± 4.6 kPa in normal tissue, with an AUC of 0.90 and a cut-off point of 44.75 kPa-values consistent with the ranges observed in our cohort.^25^ Similarly, an international meta-analysis published by Pillai *et al*. revealed that shear-wave elastography modalities (SWE and pSWE) achieve high sensitivity and specificity in differentiating benign from malignant breast lesions. However, the authors noted substantial heterogeneity among studies and therefore recommended standardizing quantitative cut-off values in each region or institution to optimize diagnostic accuracy.^26^

Our results help narrows this regional gap. In the analysed cohort, a SWE cut-off of ≥ *47.5* kPa achieved a sensitivity of 79.6% and specificity of 67.7%, whereas an elasticity index ≥ 2.25 had a sensitivity of 66.7% and specificity of 71.0%. These values fall within the ranges reported by international studies (45–60 kPa and 2.0–3.0, respectively) and suggest that, in Peruvian clinical practice, quantitative elastography parameters can be used to optimize decision-making in BI-RADS 4A and 4B lesions, where the indication for biopsy is often debatable. In this setting, low stiffness values may justify careful surveillance, whereas high values would support the recommendation for immediate biopsy, thereby helping to reduce unnecessary procedures without compromising cancer detection.

Although both elastography parameters demonstrated significant discriminative capacity in the univariate analyses, their contribution within the multivariate model showed important nuance. When conventional ultrasound features were included, BI-RADS retained the highest independent predictive strength, confirming its central role as the dominant radiologic classifier. However, once BI-RADS was removed from the model to isolate the contribution of stiffness-based metrics, SWE remained a statistically significant predictor, whereas the elastography index did not. This pattern suggests that SWE captures intrinsic biomechanical information that is not fully encompassed by morphological BI-RADS descriptors, reinforcing its value as an independent and complementary quantitative marker. These findings support the integration of SWE into breast imaging workflows as an adjunctive parameter capable of improving diagnostic confidence, especially in lesions within the BI-RADS 4A–4B range, where management decisions often remain uncertain.

The main strength of this study is that it represents one of the first Peruvian studies with a large sample size to analyse SWE, elasticity index, and BI-RADS classification jointly via an adjusted multivariate model. This integrated approach demonstrated that although BI-RADS remains the most powerful radiologic predictor, the addition of quantitative parameters enhances diagnostic discrimination and provides relevant local evidence. In this context, the values obtained in our cohort offer an initial reference for Peruvian clinical practice and represent a step toward the regional standardization of quantitative elastography.

Histopathological confirmation was available only for lesions with biopsy results recorded in our institutional electronic health record. As this was a retrospective study, some patients may have undergone biopsy at external institutions or may not have had histological verification available, which introduces potential verification bias. This limitation reflects real-world clinical practice and should be considered when interpreting diagnostic performance estimates.

All elastography examinations were performed by two experienced radiologists following a standardized institutional protocol. However, interobserver and intraobserver variability were not formally assessed due to the retrospective design of the study and the lack of stored raw elastography datasets. This limitation should be considered when interpreting the results, and future prospective studies should specifically address reproducibility.

This study makes a valuable contribution to the limited pool of Latin American evidence on quantitative breast elastography. Regional data remain scarce, and most existing SWE thresholds originate from Asian or European populations, which may differ in their breast density patterns, age distributions, and tumor biology. By generating locally validated cut-offs and demonstrating their stability, this study provides a foundational step toward establishing context-appropriate diagnostic standards for Peruvian and Latin American breast imaging practices. This aligns with international recommendations advocating for region-specific calibration of quantitative elastography systems to optimize clinical performance.

Finally, these results reinforce the need to develop national and Latin American multicenter studies to validate the proposed cut-off points and to analyse population factors such as age, breast density, and histological subtype. Establishing such standards will allow elastography to become a routine and validated tool for risk stratification and biopsy decision-making in breast cancer patients across our region.

## Conclusions

SWE and the elastography index were significantly greater in malignant lesions and were positively associated with increasing BI-RADS categories. Among the two techniques, SWE demonstrated superior sensitivity, discriminative performance, and threshold stability, underscoring its value as a complementary diagnostic tool. Its integration into routine breast ultrasound assessment may enhance risk stratification and support more informed clinical decision-making—particularly by helping reduce unnecessary biopsies in low-suspicion lesions (BI-RADS 4A) while maintaining a high standard of malignancy detection.
